# The Human Cathelicidin Antimicrobial Peptide LL-37 and Mimics are Potential Anticancer Drugs

**DOI:** 10.3389/fonc.2015.00144

**Published:** 2015-06-30

**Authors:** Kengo Kuroda, Kazuhiko Okumura, Hiroshi Isogai, Emiko Isogai

**Affiliations:** ^1^Laboratory of Animal Microbiology, Graduate School of Agricultural Science, Tohoku University, Sendai, Japan; ^2^Department of Oral and Maxillofacial Surgery, School of Dentistry, Health Sciences University of Hokkaido, Hokkaido, Japan; ^3^Animal Research Center, Sapporo Medical University, Sapporo, Japan

**Keywords:** antimicrobial peptides, anticancer, carcinogenesis, LL-37, cathelicidin

## Abstract

Antimicrobial peptides (AMPs) play a critical role in innate host defense against microbial pathogens in many organisms. The human cathelicidin, LL-37, has a net positive charge and is amphiphilic, and can eliminate pathogenic microbes directly via electrostatic attraction toward negatively charged bacterial membranes. A number of studies have shown that LL-37 participates in various host immune systems, such as inflammatory responses and tissue repair, in addition to its antibacterial properties. Moreover, recent evidence suggests that it is also involved in the regulation of cancer. Indeed, previous studies have suggested that human LL-37 is involved in carcinogenesis via multiple reporters, such as FPR2 (FPRL1), epidermal growth factor receptor, and ERBb2, although LL-37 and its fragments and analogs also show anticancer effects in various cancer cell lines. This discrepancy can be attributed to peptide-based factors, host membrane-based factors, and signal regulation. Here, we describe the association between AMPs and cancer with a focus on anticancer peptide functions and selectivity in an effort to understand potential therapeutic implications.

## Introduction

Antimicrobial peptides (AMPs) are host defense molecules of the innate immune system of all life forms (1, 2). According to the AMP database, there are over 2,000 such peptides (3). They can be divided into seven groups: (I) linear peptides; (II) cyclic peptides; (III) glycopeptides; (IV) lipoglycopeptides; (V) lipopeptides; and (VII) thiopeptides and chromopeptides. AMPs typically contain fewer than 100 amino acids and occur in many cell types. They are generally cationic and amphipathic, and homologous peptides exist in vertebrates, invertebrates, and plants.

Mammalian AMPs belong to the defensin and cathelicidin families. Defensins contain six conserved cysteine residues in their sequence and exhibit characteristic β-sheet structures stabilized by intramolecular disulfide bonds (4). Cathelicidins are characterized by a highly conserved cathelin-like prosequence and variable carboxyl-terminal sequences that correspond to the mature AMPs (5, 6). Human cationic antibacterial protein of 18 kDa (hCAP18, also called LL-37 or FALL39) is the only cathelicidin in humans, and is primarily found in the secondary granules of neutrophils (6, 7); LL-37 is released as an active domain from macrophages/monocytes and various epithelial cells (8, 9).

Antimicrobial peptides were initially identified as functional antimicrobial molecules. Recently, they have been characterized as multifunctional peptides that serve a variety of biological roles, such as immune regulation, wound healing, angiogenesis, and anticancer functions. Their anticancer activity depends on cancer types. The interactions between AMPs and cancer cells influence apoptotic or other pathways and can result in cell death. Based on their multifunctional activities, there is a growing interest in the development of AMPs as anticancer agents. Magainins, cecropins, and defensins all have anticancer effects (10). An updated list of anticancer AMPs is available in the Antimicrobial Peptide Database (APD)^1^. The anticancer activities of human AMPs have not been widely evaluated; only six members (HNP-1, HNP-2, HNP-3, hBD-1, LL-37, and granulysin) with anticancer effects are annotated in the APD. LL-37 is overexpressed in breast, ovarian, and lung cancers, but it occasionally suppresses tumorigenesis in gastric cancer (11). Considering these reports, LL-37 can be associated with dual aspects of cancer progression via various receptors, such as epidermal growth factor receptor (EGFR), FRP2, ERBb2, P2X_7_, and GAPDH, or suppression via interaction with peptide-based factors and cancer membrane components. This review is described for discussion about these functional features of AMPs including LL-37.

Our research group previously found that the modified human-derived cathelicidin-related peptide FF/CAP18 has an anti-proliferative effect on the squamous cell carcinoma-derived cell line SAS-H1 (12) and the colon cancer-derived cell line HCT-116 (13), although the detailed mechanism underlying this effect is not clear. We showed that FF/CAP18 treatment inhibits the proliferation of these cancer cell lines, and results in apoptosis and cell death. The complex involvement of LL-37 and its analogs in various cancer types requires additional studies.

## LL-37

**Figure 1:**
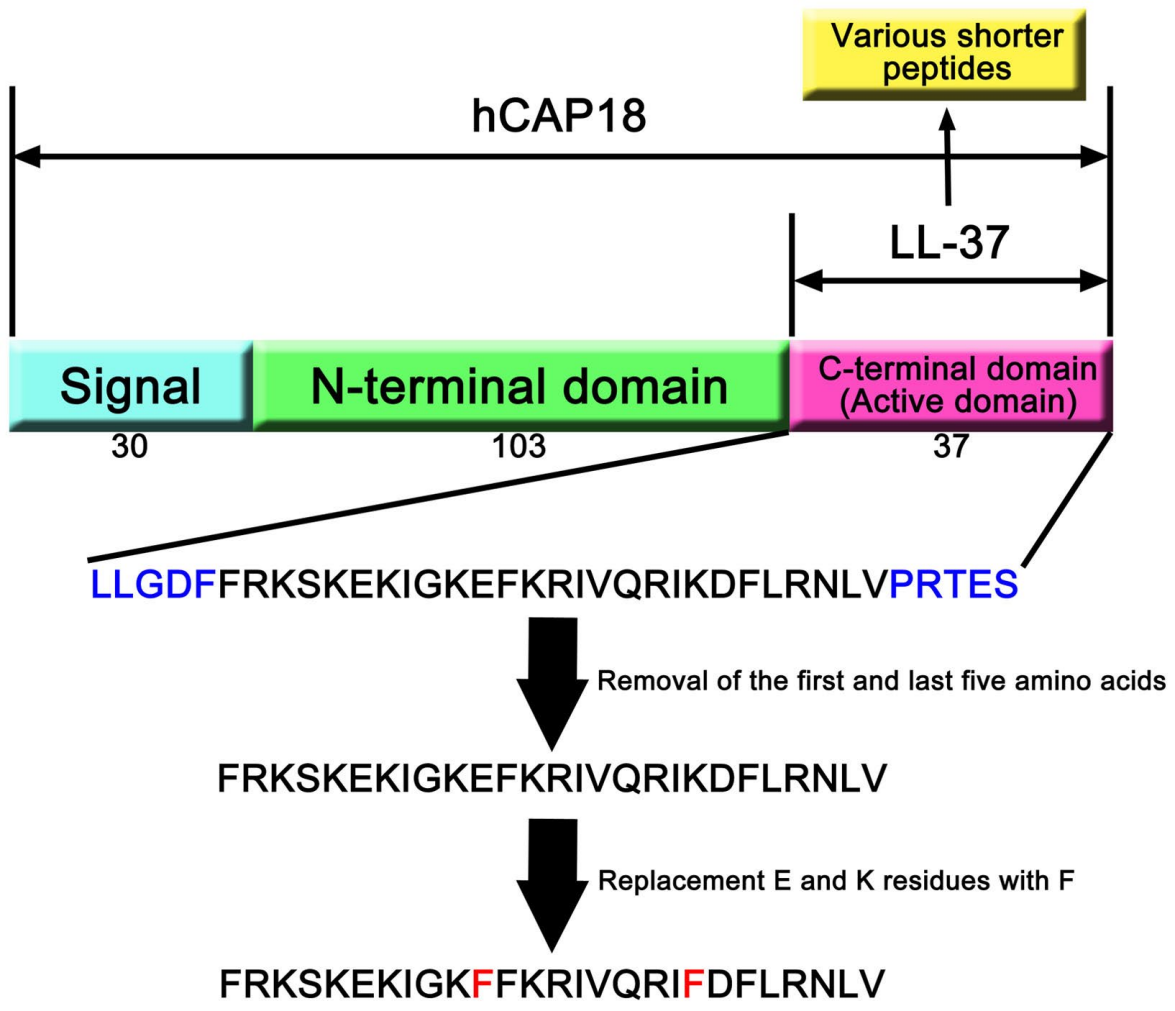
**hCAP18 and LL-37 in cathelicidin family**. The human cathelicidin hCAP18 consists of signal peptide (30 amino acids), N-terminal domain (103 amino acids), and C-terminal domain (37 amino acids). C-terminal domain shows various activities as active domain, and is called LL-37. A number of studies have revealed that shorter peptides that removed amino acids from LL-37 can show its activity. Moreover, replacement of amino acid residues can enhance its activity compared with LL-37.

Only one cathelicidin (hCAP18/LL-37) has been found in myeloid bone marrow cDNA and isolated from neutrophils (7, 14, 15). In humans, cathelicidin exons 1–4 are located on chromosome 3p21. These are transcribed as a single gene encoding CAMP (cathelicidin antimicrobial peptide), a cationic, 18-kDa pre–pro-protein, which is also referred to as hCAP18 (14, 15). As shown Figure 1, hCAP18 is characterized by an N-terminal signal peptide (30 amino acid residues), a highly conserved pro-sequence (103 amino acid residues) called the cathelin-like domain, and a mature antimicrobial peptide referred to as LL-37 (37 amino acid residues with Leu–Leu at the N-terminus) at the C-terminal domain. LL-37 is expressed in almost all tissues and organs, such as neutrophils (15), myelocytes (16), testes (7), keratinocytes (17), and saliva (18). LL-37 is the accepted family name for mature AMPs from the C-terminal region rather than the full-length protein. FALL-39 (which differs from LL-37 by two amino acids) is analogous to PR-39 discovered in cattle (7).

LL-37 has a net positive charge of +6 at a physiological pH, a hydrophobic N-terminal domain, and an α-helical conformation that is most pronounced in the presence of negatively charged lipids (6). LL-37 is produced from the C-terminal domain of the hCAP18/LL-37 precursor protein by proteolytic cleavage. hCAP18/LL-37 from specific neutrophil granules is processed to the active peptide LL-37 following exposure to serine proteases, and particularly proteinase 3 from azurophil granules after exocytosis. Proteinase 3 cleaves hCAP18/LL-37 between the alanyl and leucyl residues (6). However, proteinase 3 is only expressed in myeloid cells and not in epithelial cells. The serine proteases, stratum corneum tryptic enzyme (SCTE, kallikrein 5) and stratum corneum chymotryptic protease (SCCE, kallikrein 7), control the activation of the precursor protein hCAP18/LL-37 on the skin surface and influence further processing to smaller peptides with alternate biological activity (5). Thus, the activity of cathelicidin is controlled by enzymatic processing of the proform to a mature peptide (LL-37) and/or various short forms, such as KR20 in humans (Table 1). In addition, the prostate-derived proteinase gastricsin (pepsin C) in the presence of vaginal fluid at low pH can also process epididymal-derived hCAP18/LL-37 in seminal plasma to functionally active ALL-38 (4). The antimicrobial activity of ALL-38 against a variety of microorganisms is equal to that of LL-37.

**Table 1:** **LL-37, short peptides, and analogs**.

AMP	Sequence (type)	Net charge^a^	Antimicrobial activity	LPS-binding activity	Anticancer activity	Reference
LL-37	LLGDFFRKSKEKIGKEFKRIVQRIKDFLRNLVPRTES (original)	6	+	+	+	(7, 11, 14, 15)
LL-27 (hCAP18_109–135_)	FRKSKEKIGKEFKRIVQRIKDFLRNLV (short)	7	+	+	+	(12, 19)
LL-CAP18	FRKSKEKIGKLFKRIVQRILDFLRNLV (analog designed)	7	+ (high)	+ (high)	+ (high)	(12, 19)
FF-CAP18	FRKSKEKIGKFFKRIVQRIFDFLRNLV (analog designed)	7	+ (high)	+ (high)	+ (high)	(12, 13, 19, 20)
RK-31	RKSKEKIGKEFKRIVQRIKDFLRNLVPRTES (short)	7	+	ND	ND	(21)
KS-30	KSKEKIGKEFKRIVQRIKDFLRNLVPRTES (short)	6	+	ND	ND	(21)
KR20	KRIVQRIKDFLRNLVPRTES (short)	4	+	ND	ND	(21)
FK16	FKRIVQRIKDFLRNLV (short)	4	+	ND	+	(22)
KR12	KRIVQRIKDFLR (short)	4	+	ND	ND	(23)

*Net charge: (K + R) − (D + E)*.

## Induction of LL-37

Various stimuli can induce LL-37 (Table 2). Bacterial infection is a particularly strong inducer because AMPs are functional peptides against pathogens. *Mycobacterium tuberculosis* infection induces the expression and production of LL-37 in a variety of cells, such as epithelial cells, alveolar macrophages, neutrophils, and monocyte-derived macrophages (9). Furthermore, LPS induces strong production of LL-37. However, some studies have reported that LPS has a minimal capacity to stimulate cathelicidin production after blood mononuclear cell activation (24). This could reflect differences among cell types. It was found to be upregulated by both 1,25-hydroxyvitamin D3 and 25-hydroxyvitamin D3, and the cathelicidin gene is regulated by the vitamin D pathway in humans (25–27). Exposure to sunlight, especially ultraviolet B photons, initiates the conversion of the provitamin D3 to previtamin D3 in the skin. The second step in vitamin activation is the formation of 1,25-dihydroxyvitamin D (active vitamin D3). LL-37 can be induced by ultraviolet B irradiation and is upregulated in infected and injured skin. Gant et al. found that ultraviolet B and vitamin D may reduce the risk of several autoimmune diseases and some cancers (28). Recently, it has been reported that LL-37 is induced by various stimuli, such as short-chain fatty acids (29, 30), Zn^2+^ (31), and butyrate, which is a major metabolite produced by intestinal bacteria (32), and curcumin. Curcumin has been found to have clinical therapeutic and prevention potential for various cancers (33). Karunagaran et al. showed that curcumin-induced apoptosis mainly involves the mitochondria-mediated pathway in various cancer cells and that it inhibits proliferation of cancer cells by arresting them at various phases of the cell cycle. These effects are similar to those of LL-37 and the analogs (34). Guo et al. reported that curcumin upregulated CAMP mRNA and protein levels in U937 and HT29 cells through a vitamin D receptor-independent manner. The anticancer effect of curcumin can mediate not only direct signaling pathway but also upregulation of CAMP mRNA/the protein level and vitamin D receptor expression (35).

**Table 2:** **Known factors that induce LL-37**.

Factor	Cell types	Reference
Bacterial infection, LPS, TNF-α	A549 epithelial cells, alveolar macrophages, neutrophils, monocyte-derived macrophages, and keratinocytes	(9, 36)
Vitamin D3 (via VDR)	Neutrophil progenitors and EBV-transformed B cells, and cervical epithelial cells	(25–27)
Vitamin D3 and analogs	Myeloid leukemia, immortalized keratinocyte, and colon cancer cell lines	(37)
Short-chain fatty acids	HT-29 (colon epithelial cells) and U937 (monocytic cells)	(29, 30)
Zn^2+^	Caco-2 and intestinal epithelial cells	(31)
Butyrate	Colon, gastric, and hepatocellular cells	(32)
Curcumin	U937, HL29	(35)

## Function of LL-37 in Cancer

Cancer is a major world health problem, and it is predicted that there will be approximately 26 million new cancer cases and 17 million cancer-related deaths annually by 2030 (38). The management of cancer currently suffers from several issues. Cancer treatment strategies include radiation therapy, chemotherapy, and a combination of these, chemoradiotherapy, all of which exert cytotoxicity on cancer cells (39, 40). In addition, specific inhibitors are available, which are used for cancer therapy, such as RTK or kinase inhibitors, in the form of monoclonal antibodies or small organic molecules (41–43). Although these treatments lead to improvements in many tumor types, they can cause severe side effects and delayed neurotoxicity owing to their non-specific mechanisms, which is the first crucial matter. The second issue is the development of resistance, which is caused by a number of factors. Many conventional anticancer reagents target factors related to cancer cell growth and show poor tumor penetration, resulting in reduced sensitivity of hypoxic cells in tumors that are in a growth-arrested state (44). Furthermore, the *ABCB1* (*MDR-1*) gene can confer multidrug resistance in cancer cells via P-glycoprotein (P-gp), which belongs to the ATP-binding cassette family of transporters (45–47). P-gp expression may be low before chemotherapy; however, it is induced by chemotherapy, resulting in the transport of anticancer reagents from the cell before they interact with their intracellular targets (48). Therefore, to combat cancer, it is necessary to develop an innovative and unique therapeutic strategy. Several studies have indicated possible new targets of cancer treatment, such as the mitochondria (49), hybrid tubulin-targeting compounds (50), and anti-angiogenesis (51). However, it is generally accepted that the accumulation of oncogenes and tumor suppressor gene mutations promotes cancer development and cellular heterogeneity. High-throughput DNA sequencing data suggest that thousands of point mutations, translocations, amplifications, and deletions contribute to cancer development, and that the mutational range differs, even among tumors with identical histopathology (52). Therefore, any therapeutic strategy designed to target a single biological event or individual signaling molecules is limited with respect to its ability to improve current survival rates, and novel strategies are needed.

The identification and development of peptides with therapeutically useful anticancer potential can be an innovative strategy (53, 54). AMPs function in first-line defense against infections and exhibit potent cancer cell toxicity (55, 56). According to the APD^1^, more than 170 peptides have anticancer effects. Accumulating evidence supports the role of the human cathelicidin antimicrobial peptide LL-37 in carcinogenesis. LL-37 and its fragments and analogs show anticancer effects for various cancer cell lines. In this review, we introduce the role of AMPs, with a focus on LL-37 in human cancer in the next section.

## LL-37 as a Therapeutic Target

**Figure 2:**
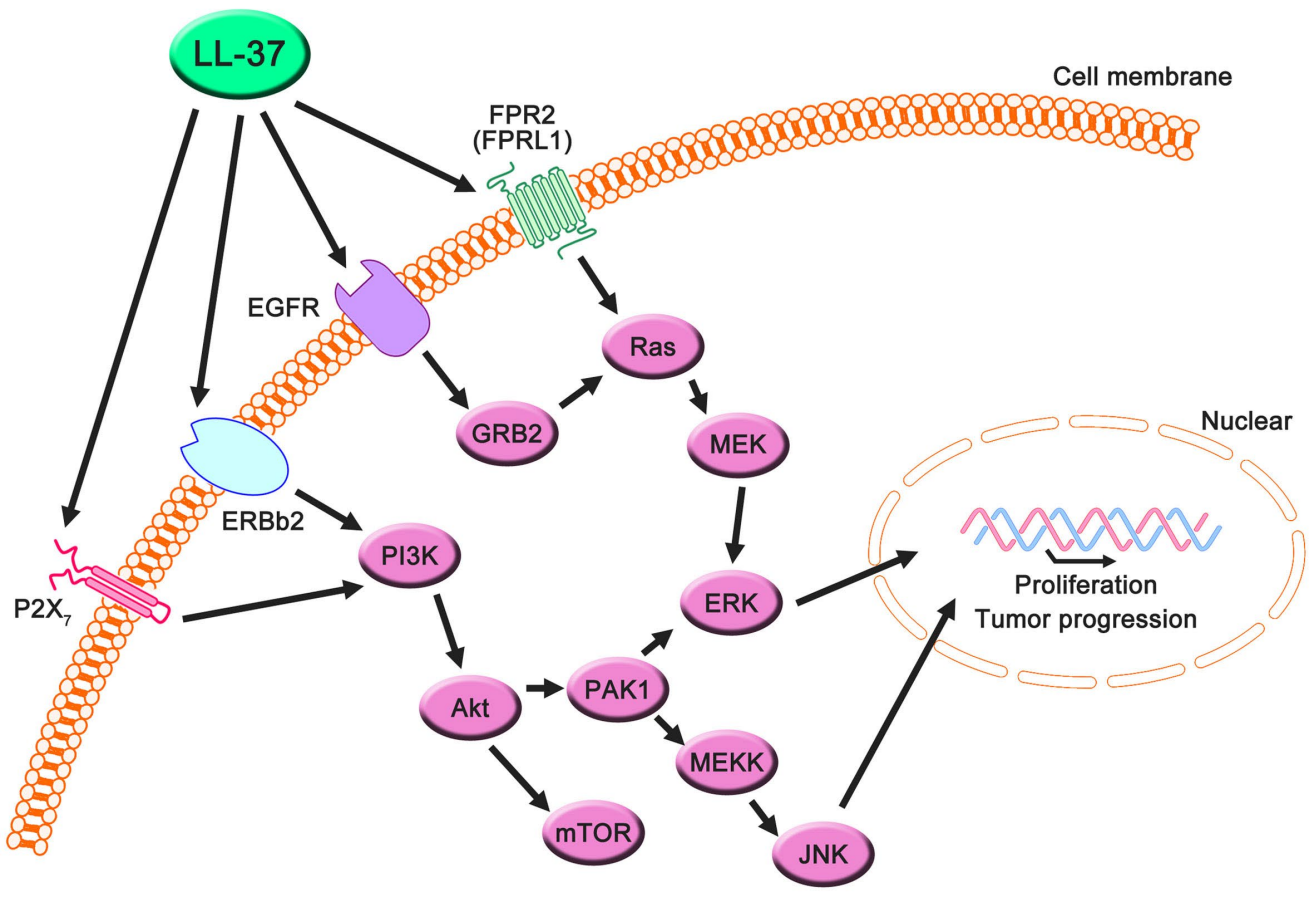
**Proposed LL-37 signaling pathways involved in cancer cell proliferation, migration, and tumor progression**. Many studies have suggested that the PI3K/Akt and MAPK/Erk signaling pathways are activated via the interaction between LL-37 and several receptors, such as FPR2 (FRPL1), EGFR, ERBb2, and P2X_7_. These signaling molecules can promote proliferation, migration, and tumor progression in cancer cells.

LL-37 is actively involved in physiological responses in eukaryotic cells, such as tissue repair and wound healing, although it was originally identified as an antimicrobial peptide. Previous studies have suggested that the possible molecular targets are involved in these effects (Table 3). LL-37 induces cell migration and downstream innate immunity via transactivation of EGFR (57, 58), and stimulates chemotaxis and angiogenesis via G-protein-coupled formyl-peptide receptor 2 (FRP2), also known as formyl-peptide receptor-like 1 (FPRL1) (8, 59, 60) (Figure 2). Based on these findings, it is not surprising that LL-37 is linked to cancer progression and metastasis. Indeed, hCAP18/LL-37 is expressed in breast cancer cells, and its production is most markedly higher in the breast epithelium of high-grade tumors than in normal mammary epithelia or low-grade tumors (>5 ng/mg total protein) (61); furthermore, FPRL1 is expressed in breast cancer (8, 62). Heilborn et al. also revealed that transgenic expression of LL-37 significantly increases proliferation in the human keratinocyte cell line (HaCaT) and HEK293. Furthermore, Weber et al. showed that mRNA expression of *hCAP18/LL-37* is strongly correlated with that of *ERBb2* and with the presence of lymph node metastasis in estrogen receptor-positive tumors from clinical samples, and LL-37 synergistically increases ErBb2 signaling (63) (Figure 2). These effects can be inhibited, suggesting the possibility of therapeutic strategies targeting LL-37. A truncated N-terminal peptide of LL-37, LL-25, inhibits LL-37 signaling and induces migration and changes in cancer cell colony morphology. Therefore, LL-37 is a putative therapeutic target to prevent progression to metastatic disease, although the detailed molecular mechanisms remain to be clarified.

**Table 3:** **Possible molecular targets of LL-37**.

Target	Cell Types	Reference
EGFR	Lung carcinoma cell line, bronchial epithelial cell line, keratinocyte	(57, 58, 64)
FRP2 (FPRL1)	293 cells stably transfected with FPRL1, eosinophils, neutrophils, umbilical vein endothelial cells, lung cancer cell lines	(8, 59, 60, 62)
ERBb2	Breast cancer cell lines	(63)
P2X_7_	Monocyte	(65)
GAPDH	Monocyte	(66)

Interestingly, these reports indicate the involvement of a receptor; AMPs generally exert effects via electrostatic interactions with the cell membrane. Several studies have shown that AMPs other than LL-37, such as SK84, a glycine-rich AMP derived from the larvae of *Drosophila virilis*, NRC-3 and NRC-7 from Atlantic flounder species, and Temporin-1CEa isolated from skin secretions of the Chinese brown frog, show breast carcinoma cytotoxicity via membrane destruction (67–69). Accordingly, the abovementioned investigations suggest the existence of signaling pathways via an LL-37-specific receptor, despite the lack of a detailed understanding of this mechanism.

Haussen et al. reported that LL-37 is expressed in human lung cancer cells (20–30 ng/mL) and acts as a growth factor (64). In this study, the EGFR signaling inhibitor AG1478 and MEK inhibitors, PD98059 and U1260, significantly inhibited LL-37-induced proliferation. Additionally, the activation of MAP kinases was detected. Thus, the effects of LL-37 on lung cancer depend on the EGFR pathway, and its effects on breast cancer depend on the downstream activation of MEK and MAP kinases (Figure 2). It is noteworthy that the concentration of LL-37 necessary to activate lung cancer cell proliferation was on the order of nanogram per milliliter, whereas the administration of 20 μg/mL LL-37 decreased rather than increased cell numbers. The LL-37 expression level in the lungs is increased during inflammatory and infectious lung diseases (70–73), and this could promote local cancer growth. Cigarette smoke induces chronic obstructive pulmonary disease, which is an inflammatory disease, and increases the risk of lung cancer development (74, 75). Recently, it was reported that mouse homolog cathelicidin-related antimicrobial peptide (CLAMP) expressed in myeloid cells promotes cigarette smoke-induced lung tumor growth by recruiting inflammatory cells (76). Therefore, there may be a strong association between human cathelicidin antimicrobial peptide LL-37, inflammation, and cancer development, and LL-37 may have unexpected positive effects for several types of cancer in normal conditions.

In prostate cancer, LL-37 is also overexpressed. *In vitro* and *in vivo* studies have demonstrated that proliferation and invasive potential decreased as a result of the targeted downregulation of CLAMP, indicating that the targeting of LL-37 in human prostate cancers could be the basis for new therapeutic strategies (77).

Coffelt et al. reported that LL-37 is significantly overexpressed in ovarian cancers relative to normal ovarian tissue and stimulates ovarian cancer cell proliferation, migration, invasion, and matrix metalloprotease secretion (1–25 μg/mL) (78). FPR2 (FPRL1) is not only involved in LL-37-stimulated cell growth but also promotes a more aggressive phenotype in ovarian cancer cells via a number of transcription factors in LL-37-FPRL1 signaling, such as cAMP response element binding protein (CREB), which may contribute to the invasive behavior of ovarian cancer cells (79). These findings indicate that LL-37-FPRL1 interactions in ovarian cancer cells are a potential target for a novel therapeutic strategy (Figure 2). The combination of CpG oligodeoxynucleotides (CpG-ODN) and LL-37 generates significant therapeutic antitumor effects in *in vivo* experiments (50–100 μg/mL) (19). Chuang et al. also observed that this combination enhances the proliferation and activation of peritoneal natural killer cells, resulting in antitumor effects. LL-37 promotes DNA translocation and can significantly increase interferon-α production in plasmacytoid dendritic cells (80); thus, it potentially delivers CpG-ODN to peritoneal immune effectors, causing potent tumor cytotoxicity. Based on these reports, LL-37 can be both a target and a candidate for therapeutic strategies for ovarian cancer.

In the last decade, P2X_7_ receptor expression and activity have been reported in several cancers (81, 82), and LL-37 is a potential ligand (65) (Figure 2). P2X_7_ triggers a range of responses including cell proliferation via the PI3K/Akt pathway (83). These findings indicate that LL-37 may promote growth via the P2X_7_ receptor in several cancers (Figure 2).

## Anticancer Effects of LL-37 as well as its Fragments and Analogs

Part of the LL-37 C-terminal domain (hCAP18_109–135_: FRKSKEKIGKEFKRIVQRIKDFLRNLV) shows anti-proliferative effects on human squamous cell carcinoma, SAS-H1, cells (20–40 μg/mL) (12). Specifically, hCAP18_109–135_ causes apoptosis via mitochondrial depolarization and DNA fragmentation but not via caspase activation. Furthermore, analog peptides with replacements of a glutamic acid residue and a lysine residue with leucine (LL/CAP18: FRKSKEKIGKLFKRIVQRILDFLRNLV) or phenylalanine (FF/CAP18: FRKSKEKIGKFFKRIVQRIFDFLRNLV) at positions 11 and 20, respectively, induce apoptotic cell death to a greater extent than did the original peptide (10–40 μg/mL). These analog peptides were designed to increase antimicrobial effects (84), which are associated with potent hydrophobic residues. This observation was based on the interactions between peptides and cancer cell membrane. These peptides, the LL-37 fragment, and its products containing amino acid substitutions can cause apoptotic cell death in cancer cells that have a more negatively charged cell membrane than in non-cancerous cells.

Several studies indicate that LL-37 and its fragments and analogs show cytotoxicity in other cancer cell types. LL-37 inhibits gastric cancer cell proliferation by the activation of bone morphogenetic protein (BMP) signaling via a proteasome-dependent mechanism (4–40 μg/mL) (22), and also induces apoptosis via the mitochondrial-associated pathway in Jurkat human T leukemia cells (25–200 μg/mL) (20). FK-16 (FKRIVQRIKDFLRNLV), which is a shorter fragment of LL-37, induces caspase-independent apoptosis and autophagy via the common p53-Bcl-2/Bax cascade in colon cancer cells (20–40 μM) (85). We have also observed that FF/CAP18 suppresses colon cancer cell proliferation via apoptotic cell death and changes metabolome levels (10–40 μg/mL) (13, 86).

**Figure 3:**
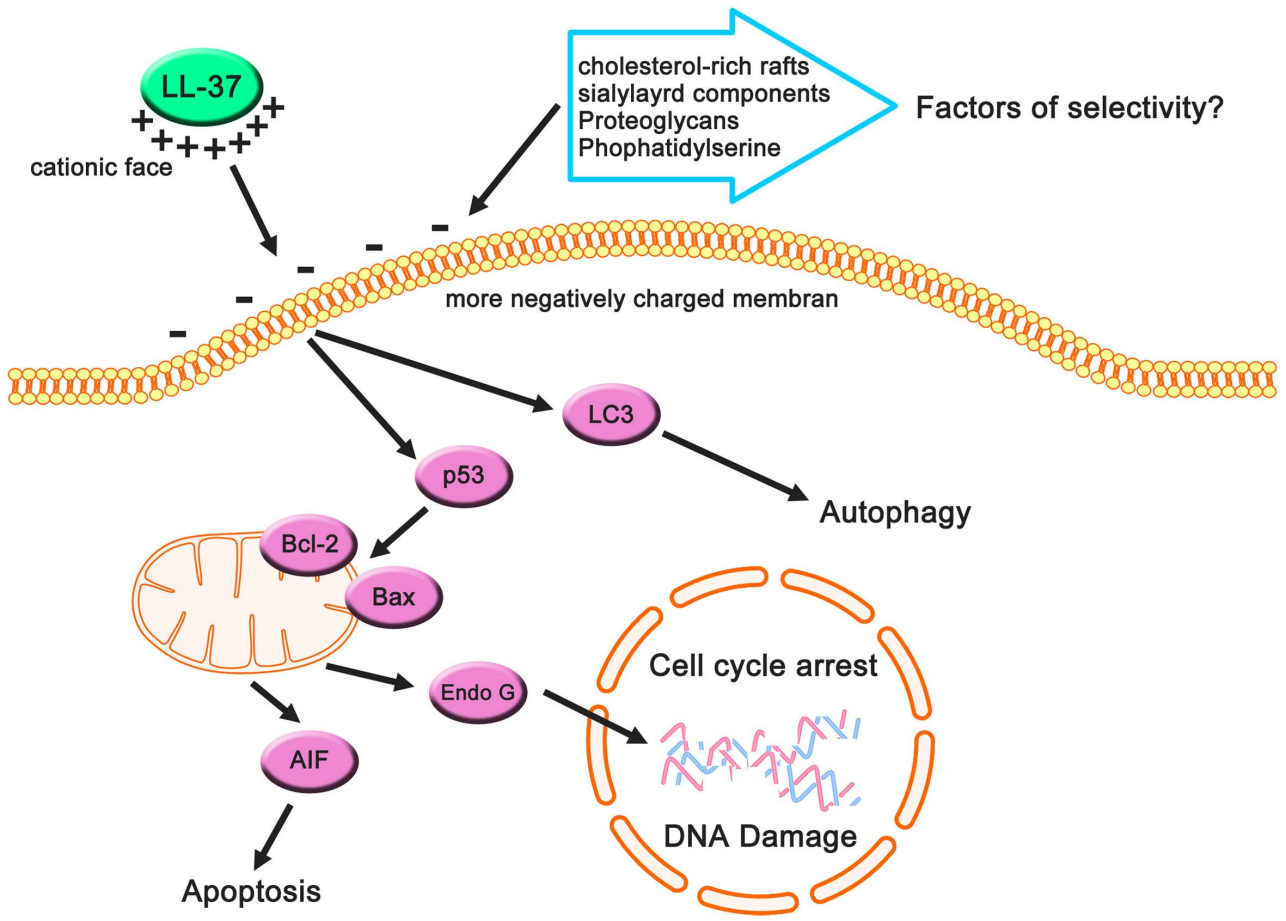
**Cancer-suppressive effects of LL-37 based on previous studies**. Cancer cells may have more negatively charged membranes compared with non-cancerous cells owing to their anionic cell components, and these components can be targets for LL-37 (which has a net positive charge). This electrostatic interaction causes apoptotic cell death, autophagy, and cell cycle arrest, resulting in the suppression of cancer cells.

In all cancer cells in which it suppressed proliferation or promoted apoptosis, autophagy, and cell cycle arrest, LL-37 expression was downregulated (87–89). In addition, cathelicidin-deficient mice exhibit increased susceptibility to azoxymethane-induced colon carcinogenesis (89). These observations suggest that LL-37 has a direct role in the suppression of tumorigenesis in several types of cancer (Figure 3), but other types of cancer may be affected by LL-37 through receptors related to proliferation or migration. These characteristics are more strongly affected by targeting the cancer membrane than by signaling induced by LL-37-receptor interactions due to the anionic cancer membrane. According to this view, the interaction between LL-37, which has a cationic charge, and the negatively charged membrane of cancer cells is extremely important with respect to the development of new therapeutic strategies, and we review the current understanding of these interactions in the next section.

## Interaction between AMPs and the Cancer Membrane

When discussing the anticancer effects of LL-37 and its fragments and analogs, it is important to consider both peptide-based factors and membrane-based factors. LL-37, one of the CAMPs, has a helical structure associated with increased peptide concentration, anions, pH, detergent, and lipids (6, 90), and interacts with the membranes of eukaryotic cancer cells. Its hydrophobicity and amphiphilicity may contribute to these interactions (91). Previous studies based on designed peptides have revealed that hydrophobicity is a critical factor in interactions between peptides and target cytoplasm membranes and the associated anticancer activity (92, 93). Moreover, anticancer peptide designed using a *de novo* approach show high specificity, i.e., they differentiate between cancerous and non-cancerous cells. It is generally recognized that amphiphilicity is a major determinant of the ability of peptides to partition the membrane; many studies have shown that changes in the amino acid distribution that disrupt the amphiphilic structure decrease the activity against bacteria or bacteria-mimic vesicles of many α-helical AMPs (94). A net positive charge is also critical for the anticancer action of AMPs (95, 96). Lysine has strong preference for anionic membranes; however, arginine, another basic residue, exhibits a strong binding affinity to both zwitterionic and anionic membranes (97). Both arginine and lysine residues have a +1 charge in neutral buffer, indicating that lysine residues could be important in determining the selectivity of cancer cell membranes, which are more anionic, relative to those of non-cancerous cells. However, these factors alone are not sufficient to predict anticancer activities (98); thus, cancer membrane-based factors may also be important.

The widely accepted model of action of AMP interactions with cancer cell membranes is that AMPs are involved in a bilayer interaction involving a membranolytic mechanism and membrane translocation for the utilization of intracellular sites. A number of studies have revealed that cancer cell membranes have distinct features relative to non-cancerous cells, such as cholesterol and a variety of anionic components (91, 96, 99). Increased cholesterol, a major sterol in eukaryotic cell membranes (100), inhibits the lytic ability of a number of α-helical peptides toward membranes of non-cancerous cells and their lipid mimics. Therefore, cholesterol plays an important role in the general lack of anticancer action on cell membranes (91, 101–103). Interestingly, some cancer cells have increased cholesterol–lipid rafts (104), indicating that the relationship between AMPs and cholesterol-lipid rafts may decrease the cancer cell toxicity (99). The formation of cholesterol-rich lipid rafts may reduce cholesterol-depleted bulk membranes harboring phosphatidylserine that are more susceptible to peptide attacks owing to increased fluidity and hence less tightly packed lipids (96). Therefore, cholesterol-rich rafts can be a key factor in the anticancer effect of AMPS (Figure 3).

The main determinant of the selectivity and toxicity of AMPs specific to cancer cells and not non-cancerous cells appears to be the overexpression of anionic membrane components, including glycoproteins, glycolipids, proteoglycans (PGs), and phospholipids on the surface of cancer cells compared with non-cancerous cells. In cancer cells, changes in the glycosylation of glycoproteins and glycolipids, including the increased expression of their terminal sialic acids (105), contribute to the selectivity of AMPs toward cancer cells (91). Several studies have shown that anticancer effects are reduced by enzymatic digestion of sialyl residues on the surface of cancer cell membranes, strongly supporting this suggestion (106, 107). Thus, sialylated components of the cancer cell membrane play an important role in the selectivity and toxicity (Figure 2). PGs, which are negatively charged, contribute to the negatively charged glycosaminoglycan side chains (108). Several studies have suggested that the expression of PGs on cancerous cell surfaces is much higher than on the surfaces of non-cancerous cells (109–111). Zwaal et al. reported that phosphatidylserine, a negatively charged phospholipid, can be exposed on the surface of the outer membrane leaflet in cancer cells (112) (Figure 2). These factors can contribute to the anticancer effects of AMPs including LL-37.

## Conclusion and Future Challenges

Despite the accumulation of scientific knowledge from a large number of studies showing that the anticancer action of LL-37 and other AMPs has potential applications for novel cancer treatment strategies, there are a few remaining challenges. In particular, its selectivity and toxicity are complicated and it will be important to consider the effects of both peptide-based and membrane-based factors. Furthermore, as we described in this review, there is a variation in the sensitivity of LL-37 among the cancer types. In breast, lung, and prostate cancers, LL-37 promotes proliferation, migration, and tumorigenesis through receptor signaling, but in other types of cancers, such as gastric cancer, colon cancer, and T-cell leukemia, it can suppress proliferation and induce apoptotic and autophagic cell death. There is no conclusive evidence to explain the opposite effects in various cancers. To resolve this issue, we may need to examine the effects of LL-37 on cancer cells from a different perspective.

It is also conceivable that LL-37 contributes to immune systems and exerts effects in combination with additional factors. Indeed, several antimicrobial agents, such as human beta defensin and LL-37, have synergistic antibacterial and anti-inflammatory activities (113–116). Therefore, LL-37 can interact with several factors to induce both positive and negative effects on cancer cells. Combination therapies with anticancer agents are a possible novel cancer treatment strategy.

Two receptors, FPR2 (55) and P2X_7_ (65), are thought to be involved in mediating the effects of LL-37 in various cell types. The glycolytic enzyme GAPDH has also been identified as a novel intracellular receptor, and is a direct binding partner for LL-37 in monocytes (66). However, the functions of these receptors including the intracellular effects mediated by LL-37 are not fully understood in cancer cells. For the application of AMPs as new therapeutic agents, it is necessary to clarify their receptor interactions and cellular mechanisms.

There are many well-known barriers to drug entry. Recently, new drug-delivery systems have been proposed (117). These approaches include mucoadhesives, viscous polymer vehicles, nanoparticles, and others. Local applications or missile treatments are possible for AMPs, irrespective of their toxicity. A novel nanocarrier was used to deliver the anticancer drug 5-fluorouracil to increase antitumor efficacy against breast cancer cells *in vitro* and *in vivo* (118). Eguchi et al. explained that magnetic anticancer drugs have the potential to greatly advance cancer chemotherapy for new theranostics and drug-delivery strategies (119). Anticancer therapy with AMPs could be successful when used in conjunction with new drug-delivery systems.

## Conflict of Interest Statement

This research was conducted in the absence of any commercial or financial relationships that could be construed as a potential conflict of interest.

## Footnotes

^1^http://aps.unmc.edu/AP/main.php

## References

[1] Zasloff M. Antimicrobial peptides of multicellular organisms. Nature (2002) 415(6870):389–95. 10.1038/415389a 11807545

[2] Zanetti M. The role of cathelicidins in the innate host defenses of mammals. Curr Issues Mol Biol (2005) 7(2):179–96. 16053249

[3] Wang G Li X Wang Z. APD2: the updated antimicrobial peptide database and its application in peptide design. Nucleic Acids Res (2009) 37(Database issue):D933–7. 10.1093/nar/gkn823 18957441 PMC2686604

[4] Sorensen OE Gram L Johnsen AH Andersson E Bangsboll S Tjabringa GS Processing of seminal plasma hCAP-18 to ALL-38 by gastricsin: a novel mechanism of generating antimicrobial peptides in vagina. J Biol Chem (2003) 278(31):28540–6. 10.1074/jbc.M301608200 12759353

[5] Yamasaki K Schauber J Coda A Lin H Dorschner RA Schechter NM Kallikrein-mediated proteolysis regulates the antimicrobial effects of cathelicidins in skin. FASEB J (2006) 20(12):2068–80. 10.1096/fj.06-6075com 17012259

[6] Johansson J Gudmundsson GH Rottenberg ME Berndt KD Agerberth B. Conformation-dependent antibacterial activity of the naturally occurring human peptide LL-37. J Biol Chem (1998) 273(6):3718–24. 10.1074/jbc.273.6.3718 9452503

[7] Agerberth B Gunne H Odeberg J Kogner P Boman HG Gudmundsson GH. FALL-39, a putative human peptide antibiotic, is cysteine-free and expressed in bone marrow and testis. Proc Natl Acad Sci U S A (1995) 92(1):195–9. 10.1073/pnas.92.1.195 7529412 PMC42844

[8] De Y Chen Q Schmidt AP Anderson GM Wang JM Wooters J LL-37, the neutrophil granule- and epithelial cell-derived cathelicidin, utilizes formyl peptide receptor-like 1 (FPRL1) as a receptor to chemoattract human peripheral blood neutrophils, monocytes, and T cells. J Exp Med (2000) 192(7):1069–74. 10.1084/jem.192.7.1069 11015447 PMC2193321

[9] Rivas-Santiago B Hernandez-Pando R Carranza C Juarez E Contreras JL Aguilar-Leon D Expression of cathelicidin LL-37 during *Mycobacterium tuberculosis* infection in human alveolar macrophages, monocytes, neutrophils, and epithelial cells. Infect Immun (2008) 76(3):935–41. 10.1128/IAI.01218-07 18160480 PMC2258801

[10] Wang G Mishra B Epand RF Epand RM. High-quality 3D structures shine light on antibacterial, anti-biofilm and antiviral activities of human cathelicidin LL-37 and its fragments. Biochim Biophys Acta (2014) 1838(9):2160–72. 10.1016/j.bbamem.2014.01.016 24463069 PMC4082733

[11] Wu WK Wang G Coffelt SB Betancourt AM Lee CW Fan D Emerging roles of the host defense peptide LL-37 in human cancer and its potential therapeutic applications. Int J Cancer (2010) 127(8):1741–7. 10.1002/ijc.25489 20521250 PMC2930073

[12] Okumura K Itoh A Isogai E Hirose K Hosokawa Y Abiko Y C-terminal domain of human CAP18 antimicrobial peptide induces apoptosis in oral squamous cell carcinoma SAS-H1 cells. Cancer Lett (2004) 212(2):185–94. 10.1016/j.canlet.2004.04.006 15279899

[13] Kuroda K Fukuda T Yoneyama H Katayama M Isogai H Okumura K Anti-proliferative effect of an analogue of the LL-37 peptide in the colon cancer derived cell line HCT116 p53+/+ and p53. Oncol Rep (2012) 28(3):829–34. 10.3892/or.2012.1876 22736062

[14] Larrick JW Lee J Ma S Li X Francke U Wright SC Structural, functional analysis and localization of the human CAP18 gene. FEBS Lett (1996) 398(1):74–80. 10.1016/S0014-5793(96)01199-4 8946956

[15] Cowland JB Johnsen AH Borregaard N. hCAP-18, a cathelin/pro-bactenecin-like protein of human neutrophil specific granules. FEBS Lett (1995) 368(1):173–6. 10.1016/0014-5793(95)00634-L 7615076

[16] Sorensen O Arnljots K Cowland JB Bainton DF Borregaard N. The human antibacterial cathelicidin, hCAP-18, is synthesized in myelocytes and metamyelocytes and localized to specific granules in neutrophils. Blood (1997) 90(7):2796–803. 9326247

[17] Frohm M Agerberth B Ahangari G Stahle-Backdahl M Liden S Wigzell H The expression of the gene coding for the antibacterial peptide LL-37 is induced in human keratinocytes during inflammatory disorders. J Biol Chem (1997) 272(24):15258–63. 10.1074/jbc.272.24.15258 9182550

[18] Murakami M Ohtake T Dorschner RA Gallo RL. Cathelicidin antimicrobial peptides are expressed in salivary glands and saliva. J Dent Res (2002) 81(12):845–50. 10.1177/154405910208101210 12454100

[19] Chuang CM Monie A Wu A Mao CP Hung CF. Treatment with LL-37 peptide enhances antitumor effects induced by CpG oligodeoxynucleotides against ovarian cancer. Hum Gene Ther (2009) 20(4):303–13. 10.1089/hum.2008.124 19272013 PMC2855250

[20] Mader JS Mookherjee N Hancock RE Bleackley RC. The human host defense peptide LL-37 induces apoptosis in a calpain- and apoptosis-inducing factor-­dependent manner involving Bax activity. Mol Cancer Res (2009) 7(5):689–702. 10.1158/1541-7786.MCR-08-0274 19435812

[21] Murakami M Lopez-Garcia B Braff M Dorschner RA Gallo RL. Postsecretory processing generates multiple cathelicidins for enhanced topical antimicrobial defense. J Immunol (2004) 172(5):3070–7. 10.4049/jimmunol.172.5.3070 14978112

[22] Wu WK Sung JJ To KF Yu L Li HT Li ZJ The host defense peptide LL-37 activates the tumor-suppressing bone morphogenetic protein signaling via inhibition of proteasome in gastric cancer cells. J Cell Physiol (2010) 223(1):178–86. 10.1002/jcp.22026 20054823

[23] Wang G. Structures of human host defense cathelicidin LL-37 and its smallest antimicrobial peptide KR-12 in lipid micelles. J Biol Chem (2008) 283(47):32637–43. 10.1074/jbc.M805533200 18818205

[24] Schauber J Dorschner RA Yamasaki K Brouha B Gallo RL. Control of the innate epithelial antimicrobial response is cell-type specific and dependent on relevant microenvironmental stimuli. Immunology (2006) 118(4):509–19. 10.1111/j.1365-2567.2006.02399.x 16895558 PMC1782325

[25] Frew L Makieva S McKinlay AT McHugh BJ Doust A Norman JE Human cathelicidin production by the cervix. PLoS One (2014) 9(8):e103434. 10.1371/journal.pone.0103434 25089904 PMC4121085

[26] Karlsson J Carlsson G Larne O Andersson M Putsep K. Vitamin D3 induces pro-LL-37 expression in myeloid precursors from patients with severe congenital neutropenia. J Leukoc Biol (2008) 84(5):1279–86. 10.1189/jlb.0607437 18703682

[27] Martineau AR Wilkinson KA Newton SM Floto RA Norman AW Skolimowska K IFN-gamma- and TNF-independent vitamin D-inducible human suppression of mycobacteria: the role of cathelicidin LL-37. J Immunol (2007) 178(11):7190–8. 10.4049/jimmunol.178.11.7190 17513768

[28] Grant WB Hypothesis – ultraviolet-B irradiance and vitamin D reduce the risk of viral infections and thus their sequelae, including autoimmune diseases and some cancers. Photochem Photobiol (2008) 84(2):356–65. 10.1111/j.1751-1097.2007.00266.x 18179620

[29] Jiang W Sunkara LT Zeng X Deng Z Myers SM Zhang G. Differential regulation of human cathelicidin LL-37 by free fatty acids and their analogs. Peptides (2013) 50:129–38. 10.1016/j.peptides.2013.10.008 24140860

[30] Zeng X Sunkara LT Jiang W Bible M Carter S Ma X Induction of porcine host defense peptide gene expression by short-chain fatty acids and their analogs. PLoS One (2013) 8(8):e72922. 10.1371/journal.pone.0072922 24023657 PMC3758276

[31] Talukder P Satho T Irie K Sharmin T Hamady D Nakashima Y Trace metal zinc stimulates secretion of antimicrobial peptide LL-37 from Caco-2 cells through ERK and p38 MAP kinase. Int Immunopharmacol (2011) 11(1):141–4. 10.1016/j.intimp.2010.10.010 21035435

[32] Schauber J Iffland K Frisch S Kudlich T Schmausser B Eck M Histone-deacetylase inhibitors induce the cathelicidin LL-37 in gastrointestinal cells. Mol Immunol (2004) 41(9):847–54. 10.1016/j.molimm.2004.05.005 15261456

[33] He Y Yue Y Zheng X Zhang K Chen S Du Z. Curcumin, inflammation, and chronic diseases: how are they linked? Molecules (2015) 20(5):9183–213. 10.3390/molecules20059183 26007179 PMC6272784

[34] Karunagaran D Rashmi R Kumar TR. Induction of apoptosis by curcumin and its implications for cancer therapy. Curr Cancer Drug Targets (2005) 5(2):117–29. 10.2174/1568009053202081 15810876

[35] Guo C Rosoha E Lowry MB Borregaard N Gombart AF. Curcumin induces human cathelicidin antimicrobial peptide gene expression through a vitamin D receptor-independent pathway. J Nutr Biochem (2013) 24(5):754–9. 10.1016/j.jnutbio.2012.04.002 22841393 PMC3485441

[36] Kim BJ Rho YK Lee HI Jeong MS Li K Seo SJ The effect of calcipotriol on the expression of human beta defensin-2 and LL-37 in cultured human ­keratinocytes. Clin Dev Immunol (2009) 2009:645898. 10.1155/2009/645898 20182640 PMC2825796

[37] Gombart AF Borregaard N Koeffler HP. Human cathelicidin antimicrobial peptide (CAMP) gene is a direct target of the vitamin D receptor and is strongly up-regulated in myeloid cells by 1,25-dihydroxyvitamin D3. FASEB J (2005) 19(9):1067–77. 10.1096/fj.04-3284com 15985530

[38] Thun MJ DeLancey JO Center MM Jemal A Ward EM. The global burden of cancer: priorities for prevention. Carcinogenesis (2010) 31(1):100–10. 10.1093/carcin/bgp263 19934210 PMC2802672

[39] Higgins GS O’Cathail SM Muschel RJ McKenna WG. Drug radiotherapy combinations: review of previous failures and reasons for future optimism. Cancer Treat Rev (2015) 41(2):105–13. 10.1016/j.ctrv.2014.12.012 25579753

[40] Urruticoechea A Alemany R Balart J Villanueva A Vinals F Capella G. Recent advances in cancer therapy: an overview. Curr Pharm Des (2010) 16(1):3–10. 10.2174/138161210789941847 20214614

[41] Ciruelos Gil EM. Targeting the PI3K/AKT/mTOR pathway in estrogen receptor-positive breast cancer. Cancer Treat Rev (2014) 40(7):862–71. 10.1016/j.ctrv.2014.03.004 24774538

[42] Vincenzi B Imperatori M Silletta M Marrucci E Santini D Tonini G. Emerging kinase inhibitors of the treatment of gastric cancer. Expert Opin Emerg Drugs (2015):1–15. 10.1517/14728214.2015.1051467 26021342

[43] Karczmarek-Borowska B Salek-Zan A. Hepatotoxicity of molecular targeted therapy. Contemp Oncol (Pozn) (2015) 19(2):87–92. 10.5114/wo.2014.43495 26034384 PMC4444439

[44] Ruan K Song G Ouyang G. Role of hypoxia in the hallmarks of human cancer. J Cell Biochem (2009) 107(6):1053–62. 10.1002/jcb.22214 19479945

[45] Ozben T. Mechanisms and strategies to overcome multiple drug resistance in cancer. FEBS Lett (2006) 580(12):2903–9. 10.1016/j.febslet.2006.02.020 16497299

[46] Goda K Bacso Z Szabo G. Multidrug resistance through the spectacle of P-glycoprotein. Curr Cancer Drug Targets (2009) 9(3):281–97. 10.2174/156800909788166493 19442049

[47] Liu FS. Mechanisms of chemotherapeutic drug resistance in cancer therapy – a quick review. Taiwan J Obstet Gynecol (2009) 48(3):239–44. 10.1016/S1028-4559(09)60296-5 19797012

[48] Thomas H Coley HM. Overcoming multidrug resistance in cancer: an update on the clinical strategy of inhibiting p-glycoprotein. Cancer Control (2003) 10(2):159–65. 12712010 10.1177/107327480301000207

[49] Pathania D Millard M Neamati N. Opportunities in discovery and delivery of anticancer drugs targeting mitochondria and cancer cell metabolism. Adv Drug Deliv Rev (2009) 61(14):1250–75. 10.1016/j.addr.2009.05.010 19716393

[50] Breen EC Walsh JJ. Tubulin-targeting agents in hybrid drugs. Curr Med Chem (2010) 17(7):609–39. 10.2174/092986710790416254 20088764

[51] Li Y Cozzi PJ. Angiogenesis as a strategic target for prostate cancer therapy. Med Res Rev (2010) 30(1):23–66. 10.1002/med.20161 19536866

[52] Parsons DW Jones S Zhang X Lin JC Leary RJ Angenendt P An integrated genomic analysis of human glioblastoma multiforme. Science (2008) 321(5897):1807–12. 10.1126/science.1164382 18772396 PMC2820389

[53] Udenigwe CC Aluko RE. Food protein-derived bioactive peptides: production, processing, and potential health benefits. J Food Sci (2012) 77(1):R11–24. 10.1111/j.1750-3841.2011.02455.x 22260122

[54] Zheng LH Wang YJ Sheng J Wang F Zheng Y Lin XK Antitumor peptides from marine organisms. Mar Drugs (2011) 9(10):1840–59. 10.3390/md9101840 22072999 PMC3210608

[55] Smolarczyk R Cichon T Szala S. [Peptides: a new class of anticancer drugs]. Postepy Hig Med Dosw (Online) (2009) 63:360–8. 19644153

[56] Schweizer F. Cationic amphiphilic peptides with cancer-selective toxicity. Eur J Pharmacol (2009) 625(1–3):190–4. 10.1016/j.ejphar.2009.08.043 19835863

[57] Tjabringa GS Aarbiou J Ninaber DK Drijfhout JW Sorensen OE Borregaard N The antimicrobial peptide LL-37 activates innate immunity at the airway epithelial surface by transactivation of the epidermal growth factor receptor. J Immunol (2003) 171(12):6690–6. 10.4049/jimmunol.171.12.6690 14662872

[58] Tokumaru S Sayama K Shirakata Y Komatsuzawa H Ouhara K Hanakawa Y Induction of keratinocyte migration via transactivation of the epidermal growth factor receptor by the antimicrobial peptide LL-37. J Immunol (2005) 175(7):4662–8. 10.4049/jimmunol.175.7.4662 16177113

[59] Tjabringa GS Ninaber DK Drijfhout JW Rabe KF Hiemstra PS. Human cathelicidin LL-37 is a chemoattractant for eosinophils and neutrophils that acts via formyl-peptide receptors. Int Arch Allergy Immunol (2006) 140(2):103–12. 10.1159/000092305 16557028

[60] Shaykhiev R Beisswenger C Kandler K Senske J Puchner A Damm T Human endogenous antibiotic LL-37 stimulates airway epithelial cell proliferation and wound closure. Am J Physiol Lung Cell Mol Physiol (2005) 289(5):L842–8. 10.1152/ajplung.00286.2004 15964896

[61] Heilborn JD Nilsson MF Jimenez CI Sandstedt B Borregaard N Tham E Antimicrobial protein hCAP18/LL-37 is highly expressed in breast cancer and is a putative growth factor for epithelial cells. Int J Cancer (2005) 114(5):713–9. 10.1002/ijc.20795 15609314

[62] Koczulla R von Degenfeld G Kupatt C Krotz F Zahler S Gloe T An angiogenic role for the human peptide antibiotic LL-37/hCAP-18. J Clin Invest (2003) 111(11):1665–72. 10.1172/JCI17545 12782669 PMC156109

[63] Weber G Chamorro CI Granath F Liljegren A Zreika S Saidak Z Human antimicrobial protein hCAP18/LL-37 promotes a metastatic phenotype in breast cancer. Breast Cancer Res (2009) 11(1):R6. 10.1186/bcr2221 19183447 PMC2687709

[64] von Haussen J Koczulla R Shaykhiev R Herr C Pinkenburg O Reimer D The host defence peptide LL-37/hCAP-18 is a growth factor for lung cancer cells. Lung Cancer (2008) 59(1):12–23. 10.1016/j.lungcan.2007.07.014 17764778

[65] Elssner A Duncan M Gavrilin M Wewers MD. A novel P2X7 receptor activator, the human cathelicidin-derived peptide LL37, induces IL-1 beta processing and release. J Immunol (2004) 172(8):4987–94. 10.4049/jimmunol.172.8.4987 15067080

[66] Mookherjee N Lippert DN Hamill P Falsafi R Nijnik A Kindrachuk J Intracellular receptor for human host defense peptide LL-37 in monocytes. J Immunol (2009) 183(4):2688–96. 10.4049/jimmunol.0802586 19605696

[67] Wang C Tian LL Li S Li HB Zhou Y Wang H Rapid cytotoxicity of antimicrobial peptide tempoprin-1CEa in breast cancer cells through membrane destruction and intracellular calcium mechanism. PLoS One (2013) 8(4):e60462. 10.1371/journal.pone.0060462 23577112 PMC3618425

[68] Hilchie AL Doucette CD Pinto DM Patrzykat A Douglas S Hoskin DW. Pleurocidin-family cationic antimicrobial peptides are cytolytic for breast carcinoma cells and prevent growth of tumor xenografts. Breast Cancer Res (2011) 13(5):R102. 10.1186/bcr3043 22023734 PMC3262215

[69] Lu J Chen ZW. Isolation, characterization and anti-cancer activity of SK84, a novel glycine-rich antimicrobial peptide from Drosophila virilis. Peptides (2010) 31(1):44–50. 10.1016/j.peptides.2009.09.028 19799950

[70] Xiao W Hsu YP Ishizaka A Kirikae T Moss RB. Sputum cathelicidin, urokinase plasminogen activation system components, and cytokines discriminate cystic fibrosis, COPD, and asthma inflammation. Chest (2005) 128(4):2316–26. 10.1378/chest.128.4.2316 16236890

[71] Soong LB Ganz T Ellison A Caughey GH. Purification and characterization of defensins from cystic fibrosis sputum. Inflamm Res (1997) 46(3):98–102. 10.1007/s000110050114 9098722

[72] Schaller-Bals S Schulze A Bals R. Increased levels of antimicrobial peptides in tracheal aspirates of newborn infants during infection. Am J Respir Crit Care Med (2002) 165(7):992–5. 10.1164/ajrccm.165.7.200110-020 11934727

[73] Agerberth B Grunewald J Castanos-Velez E Olsson B Jornvall H Wigzell H Antibacterial components in bronchoalveolar lavage fluid from healthy individuals and sarcoidosis patients. Am J Respir Crit Care Med (1999) 160(1):283–90. 10.1164/ajrccm.160.1.9807041 10390413

[74] Young RP Hopkins RJ Christmas T Black PN Metcalf P Gamble GD. COPD prevalence is increased in lung cancer, independent of age, sex and smoking history. Eur Respir J (2009) 34(2):380–6. 10.1183/09031936.00144208 19196816

[75] Papi A Casoni G Caramori G Guzzinati I Boschetto P Ravenna F COPD increases the risk of squamous histological subtype in smokers who develop non-small cell lung carcinoma. Thorax (2004) 59(8):679–81. 10.1136/thx.2003.018291 15282388 PMC1747095

[76] Li D Beisswenger C Herr C Schmid RM Gallo RL Han G Expression of the antimicrobial peptide cathelicidin in myeloid cells is required for lung tumor growth. Oncogene (2014) 33(21):2709–16. 10.1038/onc.2013.248 23812430

[77] Hensel JA Chanda D Kumar S Sawant A Grizzle WE Siegal GP LL-37 as a therapeutic target for late stage prostate cancer. Prostate (2011) 71(6):659–70. 10.1002/pros.21282 20957672 PMC3025071

[78] Coffelt SB Waterman RS Florez L Honer zu Bentrup K Zwezdaryk KJ Tomchuck SL Ovarian cancers overexpress the antimicrobial protein hCAP-18 and its derivative LL-37 increases ovarian cancer cell proliferation and invasion. Int J Cancer (2008) 122(5):1030–9. 10.1002/ijc.23186 17960624

[79] Coffelt SB Tomchuck SL Zwezdaryk KJ Danka ES Scandurro AB. Leucine leucine-37 uses formyl peptide receptor-like 1 to activate signal transduction pathways, stimulate oncogenic gene expression, and enhance the invasiveness of ovarian cancer cells. Mol Cancer Res (2009) 7(6):907–15. 10.1158/1541-7786.MCR-08-0326 19491199 PMC2755540

[80] Lande R Gregorio J Facchinetti V Chatterjee B Wang YH Homey B Plasmacytoid dendritic cells sense self-DNA coupled with antimicrobial peptide. Nature (2007) 449(7162):564–9. 10.1038/nature06116 17873860

[81] Adinolfi E Amoroso F Giuliani AL. P2X7 receptor function in bone-related cancer. J Osteoporos (2012) 2012:637863. 10.1155/2012/637863 22970409 PMC3431089

[82] Adinolfi E Capece M Amoroso F De Marchi E Franceschini A. Emerging roles of P2X receptors in cancer. Curr Med Chem (2015) 22(7):878–90. 10.2174/0929867321666141012172913 25312206

[83] Amoroso F Capece M Rotondo A Cangelosi D Ferracin M Franceschini A The P2X7 receptor is a key modulator of the PI3K/GSK3beta/VEGF signaling network: evidence in experimental neuroblastoma. Oncogene (2015). 10.1038/onc.2014.444 25619831

[84] Isogai E Isogai H Matuo K Hirose K Kowashi Y Okumuara K Sensitivity of genera *Porphyromonas* and *Prevotella* to the bactericidal action of C-terminal domain of human CAP18 and its analogues. Oral Microbiol Immunol (2003) 18(5):329–32. 10.1034/j.1399-302X.2003.00083.x 12930528

[85] Ren SX Shen J Cheng AS Lu L Chan RL Li ZJ FK-16 derived from the anticancer peptide LL-37 induces caspase-independent apoptosis and autophagic cell death in colon cancer cells. PLoS One (2013) 8(5):e63641. 10.1371/journal.pone.0063641 23700428 PMC3659029

[86] Kuroda K Fukuda T Isogai H Okumura K Krstic-Demonacos M Isogai E. Antimicrobial peptide FF/CAP18 induces apoptotic cell death in HCT116 colon cancer cells via changes in the metabolic profile. Int J Oncol (2015) 46(4):1516–26. 10.3892/ijo.2015.2887 25672949 PMC4356497

[87] Hase K Murakami M Iimura M Cole SP Horibe Y Ohtake T Expression of LL-37 by human gastric epithelial cells as a potential host defense mechanism against *Helicobacter pylori*. Gastroenterology (2003) 125(6):1613–25. 10.1053/j.gastro.2003.08.028 14724813

[88] Yang YH Zheng GG Li G Zhang B Song YH Wu KF. Expression of LL-37/hCAP-18 gene in human leukemia cells. Leuk Res (2003) 27(10):947–50. 10.1016/S0145-2126(03)00020-1 12860015

[89] Ren SX Cheng AS To KF Tong JH Li MS Shen J Host immune defense peptide LL-37 activates caspase-independent apoptosis and suppresses colon cancer. Cancer Res (2012) 72(24):6512–23. 10.1158/0008-5472.CAN-12-2359 23100468 PMC3910284

[90] Oren Z Lerman JC Gudmundsson GH Agerberth B Shai Y. Structure and organization of the human antimicrobial peptide LL-37 in phospholipid membranes: relevance to the molecular basis for its non-cell-selective activity. Biochem J (1999) 341(Pt 3):501–13. 10.1042/0264-6021:3410501 10417311 PMC1220385

[91] Dennison SR Whittaker M Harris F Phoenix DA. Anticancer alpha-helical peptides and structure/function relationships underpinning their interactions with tumour cell membranes. Curr Protein Pept Sci (2006) 7(6):487–99. 10.2174/138920306779025611 17168782

[92] Chen Y Mant CT Farmer SW Hancock RE Vasil ML Hodges RS. Rational design of alpha-helical antimicrobial peptides with enhanced activities and specificity/therapeutic index. J Biol Chem (2005) 280(13):12316–29. 10.1074/jbc.M413406200 15677462 PMC1393284

[93] Huang YB Wang XF Wang HY Liu Y Chen Y. Studies on mechanism of action of anticancer peptides by modulation of hydrophobicity within a defined structural framework. Mol Cancer Ther (2011) 10(3):416–26. 10.1158/1535-7163.MCT-10-0811 21252288

[94] Lorin A Noel M Provencher ME Turcotte V Masson C Cardinal S Revisiting peptide amphiphilicity for membrane pore formation. Biochemistry (2011) 50(43):9409–20. 10.1021/bi201335t 21942823

[95] Al-Benna S Shai Y Jacobsen F Steinstraesser L. Oncolytic activities of host defense peptides. Int J Mol Sci (2011) 12(11):8027–51. 10.3390/ijms12118027 22174648 PMC3233454

[96] Riedl S Zweytick D Lohner K. Membrane-active host defense peptides – challenges and perspectives for the development of novel anticancer drugs. Chem Phys Lipids (2011) 164(8):766–81. 10.1016/j.chemphyslip.2011.09.004 21945565 PMC3220766

[97] Yang ST Shin SY Lee CW Kim YC Hahm KS Kim JI. Selective cytotoxicity following Arg-to-Lys substitution in tritrpticin adopting a unique amphipathic turn structure. FEBS Lett (2003) 540(1–3):229–33. 10.1016/S0014-5793(03)00266-7 12681513

[98] Dennison SR Harris F Bhatt T Singh J Phoenix DA. A theoretical analysis of secondary structural characteristics of anticancer peptides. Mol Cell Biochem (2010) 333(1–2):129–35. 10.1007/s11010-009-0213-3 19629645

[99] Harris F Dennison SR Singh J Phoenix DA. On the selectivity and efficacy of defense peptides with respect to cancer cells. Med Res Rev (2013) 33(1):190–234. 10.1002/med.20252 21922503

[100] Simons K Ikonen E How cells handle cholesterol. Science (2000) 290(5497):1721–6. 10.1126/science.290.5497.1721 11099405

[101] Matsuzaki K Sugishita K Fujii N Miyajima K. Molecular basis for membrane selectivity of an antimicrobial peptide, magainin 2. Biochemistry (1995) 34(10):3423–9. 10.1021/bi00010a034 7533538

[102] Wojcik C Sawicki W Marianowski P Benchaib M Czyba JC Guerin JF. Cyclodextrin enhances spermicidal effects of magainin-2-amide. Contraception (2000) 62(2):99–103. 10.1016/S0010-7824(00)00143-8 11102594

[103] Steiner H Andreu D Merrifield RB. Binding and action of cecropin and cecropin analogues: antibacterial peptides from insects. Biochim Biophys Acta (1988) 939(2):260–6. 10.1016/0005-2736(88)90069-7 3128324

[104] Li YC Park MJ Ye SK Kim CW Kim YN. Elevated levels of cholesterol-rich lipid rafts in cancer cells are correlated with apoptosis sensitivity induced by cholesterol-depleting agents. Am J Pathol (2006) 168(4):1107–18. 10.2353/ajpath.2006.050959 16565487 PMC1606567

[105] Varki A Sialic acids in human health and disease. Trends Mol Med (2008) 14(8):351–60. 10.1016/j.molmed.2008.06.002 18606570 PMC2553044

[106] Fredman P Hedberg K Brezicka T. Gangliosides as therapeutic targets for cancer. BioDrugs (2003) 17(3):155–67. 10.2165/00063030-200317030-00002 12749752

[107] Ohyama C. Glycosylation in bladder cancer. Int J Clin Oncol (2008) 13(4):308–13. 10.1007/s10147-008-0809-8 18704630

[108] Schaefer L Schaefer RM. Proteoglycans: from structural compounds to signaling molecules. Cell Tissue Res (2010) 339(1):237–46. 10.1007/s00441-009-0821-y 19513755

[109] Iozzo RV Sanderson RD. Proteoglycans in cancer biology, tumour microenvironment and angiogenesis. J Cell Mol Med (2011) 15(5):1013–31. 10.1111/j.1582-4934.2010.01236.x 21155971 PMC3633488

[110] Asimakopoulou AP Theocharis AD Tzanakakis GN Karamanos NK. The biological role of chondroitin sulfate in cancer and chondroitin-based anticancer agents. In vivo (2008) 22(3):385–9. 18610752

[111] Koo CY Sen YP Bay BH Yip GW. Targeting heparan sulfate proteoglycans in breast cancer treatment. Recent Pat Anticancer Drug Discov (2008) 3(3):151–8. 10.2174/157489208786242278 18991783

[112] Zwaal RF Comfurius P Bevers EM. Surface exposure of phosphatidylserine in pathological cells. Cell Mol Life Sci (2005) 62(9):971–88. 10.1007/s00018-005-4527-3 15761668 PMC11924510

[113] Chen X Niyonsaba F Ushio H Okuda D Nagaoka I Ikeda S Synergistic effect of antibacterial agents human beta-defensins, cathelicidin LL-37 and lysozyme against *Staphylococcus aureus* and *Escherichia coli*. J Dermatol Sci (2005) 40(2):123–32. 10.1016/j.jdermsci.2005.03.014 15963694

[114] Nagaoka I Hirota S Yomogida S Ohwada A Hirata M. Synergistic actions of antibacterial neutrophil defensins and cathelicidins. Inflamm Res (2000) 49(2):73–9. 10.1007/s000110050561 10738945

[115] Midorikawa K Ouhara K Komatsuzawa H Kawai T Yamada S Fujiwara T *Staphylococcus aureus* susceptibility to innate antimicrobial peptides, beta-defensins and CAP18, expressed by human keratinocytes. Infect Immun (2003) 71(7):3730–9. 10.1128/IAI.71.7.3730-3739.2003 12819054 PMC162002

[116] Maisetta G Batoni G Esin S Luperini F Pardini M Bottai D Activity of human beta-defensin 3 alone or combined with other antimicrobial agents against oral bacteria. Antimicrob Agents Chemother (2003) 47(10):3349–51. 10.1128/AAC.47.10.3349-3351.2003 14506056 PMC201125

[117] Kompella UB Kadam RS Lee VH Recent advances in ophthalmic drug delivery. Ther Deliv (2010) 1(3):435–56. 10.4155/tde.10.40 21399724 PMC3051398

[118] Yuan Z Qu X Wang Y Zhang DY Luo JC Jia N Enhanced antitumor efficacy of 5-fluorouracil loaded methoxy poly(ethylene glycol)-poly(lactide) nanoparticles for efficient therapy against breast cancer. Colloids Surf B Biointerfaces (2015) 128:489–97. 10.1016/j.colsurfb.2015.02.048 25779606

[119] Eguchi H Umemura M Kurotani R Fukumura H Sato I Kim JH A magnetic anti-cancer compound for magnet-guided delivery and magnetic resonance imaging. Sci Rep (2015) 5:9194. 10.1038/srep09194 25779357 PMC4361848

